# *Curly* Encodes Dual Oxidase, Which Acts with Heme Peroxidase Curly Su to Shape the Adult *Drosophila* Wing

**DOI:** 10.1371/journal.pgen.1005625

**Published:** 2015-11-20

**Authors:** Thomas Ryan Hurd, Feng-Xia Liang, Ruth Lehmann

**Affiliations:** HHMI and Kimmel Center for Biology and Medicine of the Skirball Institute, Department of Cell Biology, New York University School of Medicine, New York, New York, United States of America; Cancer Research UK, UNITED KINGDOM

## Abstract

*Curly*, described almost a century ago, is one of the most frequently used markers in *Drosophila* genetics. Despite this the molecular identity of *Curly* has remained obscure. Here we show that *Curly* mutations arise in the gene *dual oxidase (duox)*, which encodes a reactive oxygen species (ROS) generating NADPH oxidase. Using *Curly* mutations and RNA interference (RNAi), we demonstrate that Duox autonomously stabilizes the wing on the last day of pupal development. Through genetic suppression studies, we identify a novel heme peroxidase, Curly Su (Cysu) that acts with Duox to form the wing. Ultrastructural analysis suggests that Duox and Cysu are required in the wing to bond and adhere the dorsal and ventral cuticle surfaces during its maturation. In *Drosophila*, Duox is best known for its role in the killing of pathogens by generating bactericidal ROS. Our work adds to a growing number of studies suggesting that Duox’s primary function is more structural, helping to form extracellular and cuticle structures in conjunction with peroxidases.

## Author Summary

Fruit fly geneticists rely on a handful of dominant mutations that modify adult morphology in a way that is easy to spot, like changing the shape of the fly’s wings, eyes or bristles. One of the first such mutants identified in the early days of fly genetics and to this day likely the most widely used mutation, is *Curly*, which causes an upward curvature in the adult wings. Despite its importance as a marker, the genetic cause of *Curly* has remained unknown. Here, we reveal that *Curly* mutations occur in the gene *duox*, which encodes a ROS-generating enzyme. ROS once thought to be merely harmful by-products of metabolism, can also have beneficial purposes. Here we provide evidence that Duox generates ROS to help form and stabilize the wings of fruit flies. Furthermore, we identify a second enzyme, Cysu, which uses the ROS generated by Duox to crosslink proteins in the wing, thereby stabilizing and shaping its structure. Duox occurs in numerous organisms, including humans and fulfills a number of other functions, in particular in immunity and pathogen defense. With this new knowledge, *Curly* mutations will provide an excellent tool to study and understand the roles Duox plays in a variety of biological contexts.

## Introduction

Over 90 years ago, Lenore Ward first described a dominant mutation, *Curly*, that causes the wings of *Drosophila melanogaster* to bend upwards [1]. Since then, *Curly* has become a ubiquitous second chromosomal marker used by *Drosophila* geneticists on a daily basis to follow and track mutations. Despite its widespread use, how *Curly* mutations dominantly alter wing curvature has remained obscure. Waddington first proposed that *Curly* causes an unequal contraction of the dorsal and ventral wing surfaces during the drying period shortly after flies emerge from their pupal cases [2, 3]. Others have subsequently demonstrated that comparable alterations in wing curvature can be caused by differential growth of the dorsal and ventral epithelia [4]. Irrespective of the mechanism, that similar wing phenotypes have been described for *D*. *pseudoobscura* and *D*. *montium* mutants [5] suggests the underlying cause of curly wing formation is evolutionarily conserved among Drosophilids [2]. The major factor limiting our understanding of *Curly’s* function in wing morphogenesis, however, is the fact that its molecular identity has remained unknown.

In this manuscript we uncover the long unknown molecular nature of *Curly*. We show that mutations in the gene *duox* cause the *Curly* wing phenotype. Duox is a member of a highly conserved group of transmembrane proteins collectively referred to as NADPH oxidases. These enzymes function to transfer electrons across biological membranes to generate ROS by transferring electrons from NADPH to oxygen through flavin adenine dinucleotide (FAD) and heme cofactors [6]. Several biological functions have been described for Duox. Perhaps the best studied of these in *Drosophila* is its role in host defense where it is thought to generate ROS to kill pathogens [7]. However, Duox also plays an important role in providing ROS, specifically hydrogen peroxide, for heme peroxidases to catalyze the formation of covalent bonds between biomolecules. In mammals, Duox generates hydrogen peroxide for thyroid peroxidase to catalyze the iodination and crosslinking of tyrosine residues in the formation of thyroid hormones [8, 9]. Duox is also expressed in tissues other than the thyroid, such as the gastrointestinal tract, where its function is less clear [6]. In insects, worms and sea urchins, Duox participates in the formation of extracellular structures through the crosslinking of tyrosine residues [10–12]. Indeed, instead of its function in generating bactericidal ROS, the tyrosine crosslinking activity of Duox may be the primary ancestral function, as it appears to be conserved across phyla.

Here we show that specific mutations in the NADPH binding-domain encoding region of *duox* cause a *Curly* wing phenotype. Using *Curly*, we demonstrate that *duox* is required during the last day of pupal development to stabilize the wing. Furthermore, through suppression experiments, we identify a novel heme peroxidase, Curly Su (Cysu), that works with *duox* to adhere the dorsal surface of the wing to the ventral one. Uncovering the molecular identity of *Curly* not only provides an entry point for the functional understanding of this prominent wing mutant phenotype, but also will allow for the discovery of novel *duox* interacting genes and regulators through unbiased genetic screens. Only through these approaches can we hope to understand the precise molecular function of Duox in the myriad biological processes in which it is involved.

## Results

### *Curly* mutations occur within *duox*

In the course of genetically following a loss-of-function mutation in the gene *duox*, *duox* ^*KG07745*^, using the standard *Drosophila* genetic tool *Curly of Oster* (*CyO)* balancer, we noticed that we were unable to recover progeny containing both *duox* ^*KG07745*^ and *CyO*. Since a) *duox* and *Curly* mutations fail to complement one another and b) *Curly* roughly maps to 23A4-23B2 [13], the chromosomal region containing *duox*, we wondered whether *Curly* might be an allele of *duox*.

**Fig 1:**
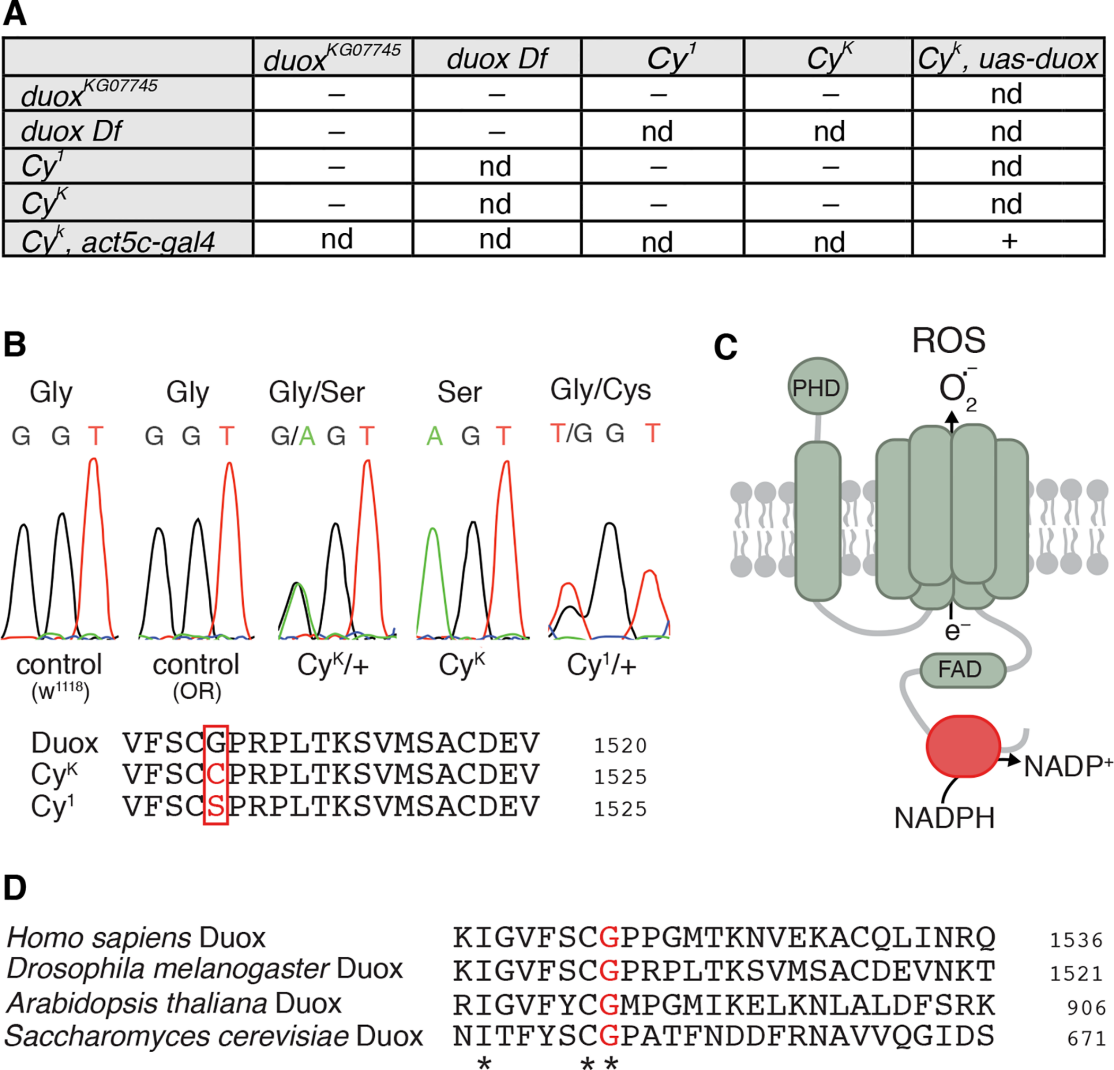
*Curly* is an allele of *duox*. (A) *duox* mutations fail to complement *Curly* mutations. *Curly* mutations are lethal as homozygotes and as transheterozygotes with a *duox* mutation or a deficiency uncovering the *duox* locus. Ubiquitous expression of *duox* restores viability to homozygous *Curly* mutants.– = not viable, + = viable, and nd = not determined. (B) *Curly* mutations cause a single amino acid change of a conserved glycine to either serine or cysteine for *Cy* ^*K*^ or *Cy* ^*1*^, respectively. (C) Cartoon of Duox. In red is the NADPH binding domain. PHD stands for peroxidase homology domain. (D) The conserved glycine residue mutated in *Curly* is an essential amino acid in the NADPH binding domain of Duox.

Balancer chromosomes contain numerous inversions and chromosomal aberrations. To exclude the possibility that our inability to recover progeny was due to some other lesion in the 23A4-23B2 region of *CyO*, we crossed *duox* ^*KG07745*^ to various *Curly* mutations not associated with *CyO* [1, 13, 14]. Consistent with our earlier results, all the *Curly* alleles tested failed to complement *duox* ^*KG07745*^, suggesting that *Curly* mutations indeed reside within the *duox* locus (Fig 1A). To provide conclusive evidence, however, that *duox* and *Curly* are one and the same, we expressed *duox* ubiquitously in a *Curly* mutant background. Ubiquitous expression of *duox* restored viability, allowing recovery of homozygous *Curly* mutants (Fig 1A). These results strongly suggest that the *Curly* phenotype is due to mutations in the *duox* gene.

### *Curly* mutations occur in the NADPH-binding pocket encoding region of *duox*

To precisely determine how *Curly* mutations alter *duox’s* nucleotide sequence, we sequenced *duox* in two *Curly* mutants: *Cy* ^*K*^, which was previously generated using ethyl methanesulfonate [14]; and *Cy* ^*1*^, the original spontaneous mutation identified by Ward [1]. Remarkably, we found that the same nucleotide was mutated in both *Cy* ^*K*^ and *Cy* ^*1*^ (Fig 1B). This single nucleotide mutation resulted in the conversion of a conserved glycine (number 1505) in the NADPH binding domain of Duox to a serine in *Cy* ^*K*^ and to a cysteine in *Cy* ^*1*^ (Fig 1C, red). Among the residues within the NADPH binding pocket of Duox, glycine 1505 is extraordinarily well conserved. It is present in all known NADPH oxidases from yeast to humans (Fig 1D), suggesting that it is functionally important. Therefore a conversion of a conserved glycine to a polar amino acid specifically in the NADPH binding pocket of Duox causes *Curly*. Taken together, our results demonstrate, after more than 90 years since its discovery, that the *Curly* phenotype is due to mutations within the *duox* gene.

### *Curly* is likely a neomorphic allele of *duox*

Mutations in the NADPH binding encoding region of *duox* cause a *Curly* phenotype, but how exactly do they influence Duox’s function? From complementation experiments, it is clear that *Curly* mutations reduce Duox’s normal function as they are not viable in combination with either a loss-of-function *duox* mutation or a deficiency uncovering the *duox* locus (Fig 1A). However, *Curly* mutants also act dominantly, causing the wings of *Curly* flies to bend upwards (hence the name) in contrast to the straight wings of wild-type or *duox* heterozygous flies. One possible explanation is that *Curly* mutations act in opposition to Duox’s normal activity as dominant negatives or antimorphs. This, however, is unlikely because ubiquitous overexpression of wild-type Duox in a *Curly* mutant background failed to restore normal wing shape (S1 Fig). Another possibility is that *Curly* mutations increase the normal function of Duox either by increasing expression or constitutively activating Duox. However, this too seems implausible because removing wild-type Duox in *Curly* mutants worsened (causing lethality) rather than ameliorated the viability phenotype (Fig 1A). Instead, the most likely explanation is that *Curly* mutations are neomorphic, causing a dominant gain-of-function in Duox that is different from its normal function.

**Fig 2:**
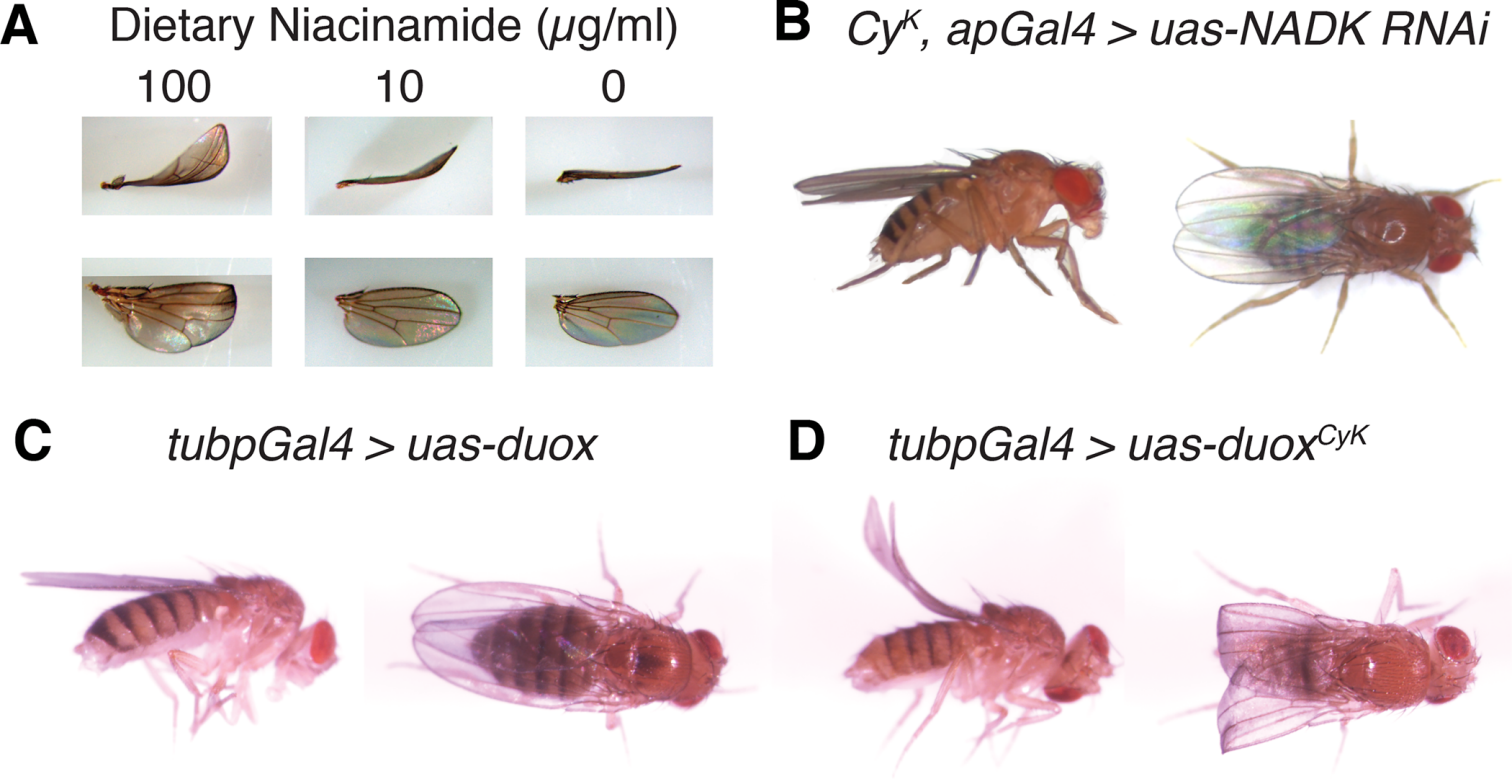
Curly is likely a neomorphic allele of duox. (A) Reduction of dietary niacinamide causes a dose-dependent decrease in the expressivity of the *Curly* wing phenotype. Flies were raised on food containing different concentrations of niacinamide. (B) Knockdown of the NAD^+^ kinase, CG6145, with the wing driver apGal4 suppresses the *Curly* wing phenotype. Knockdown of CG6145 with apGal4 alone did not cause a wing phenotype. (C) and (D) Ubiquitous expression of *duox* ^*CyK*^, but not *duox*, causes a *Curly* wing phenotype. *UAS-duox* and *UAS-duox* ^*CyK*^ were expressed ubiquitously using the *tubpGal4* driver.

To further investigate the potential neomorphic nature of *Curly* mutations, we tested whether *Curly* mutations require NADPH, the substrate of Duox, to generate a wing phenotype. In the first instance, we attempted to alter the endogenous levels of NADPH by reducing the amount of niacinamide, a precursor of NADPH, in the diet of *Curly* flies. Interestingly, we found that a reduction of dietary niacinamide caused a dose-dependent decrease in the expressivity of the wing phenotype in *Curly* mutants (Fig 2A). To test this genetically, we next knocked down *CG6145*, which encodes a NAD^+^ kinase that phosphorylates NAD^+^ to generate NADP^+^ [15]. Specific knockdown of CG6145 in the wing using *apterous-gal4 (apGal4)* strongly suppressed the *Curly* wing phenotype (Fig 2B). Together these results suggest that *Curly* mutants require NADP^+^ and/or NADPH in order to cause changes in wing curvature. Therefore, not only do *Curly* mutations reduce Duox’s normal function, they also endow it with a new function that requires sufficient substrate to dominantly alter wing shape.

### Duox is required autonomously in the wing during the last day of development to stabilize the wing

Having determined that *Curly* is most likely a neomophoric allele of *duox*, we decided to use *Curly* mutations to explore *duox’s* function *in vivo* in the wing. To do this, we generated a mutant form of *duox*, henceforth referred to as *duox* ^*CyK*^, in which glycine 1505 was mutated to a serine, as in the *Cy* ^*K*^ mutant. In order to express *duox* ^*CyK*^ conditionally we fused it to an Upstream Activating Sequence (UAS) element so that we could control its expression in a time-dependent and tissue-specific manner using the Gal4 system [16]. To demonstrate that this transgene was functional and able to recreate the *Curly* phenotype, we expressed it ubiquitously throughout the fly using *tubpGal4* [17]. Indeed, ubiquitous expression of *duox* ^*CyK*^, but not wild-type *duox*, resulted in upward bent wings resembling those of *Cy* ^*K*^ mutants (Fig 2C and 2D). This not only demonstrates that the *duox* ^*CyK*^ transgene is functional, but also provides further evidence that *Curly* is an allele of *duox*.

**Fig 3:**
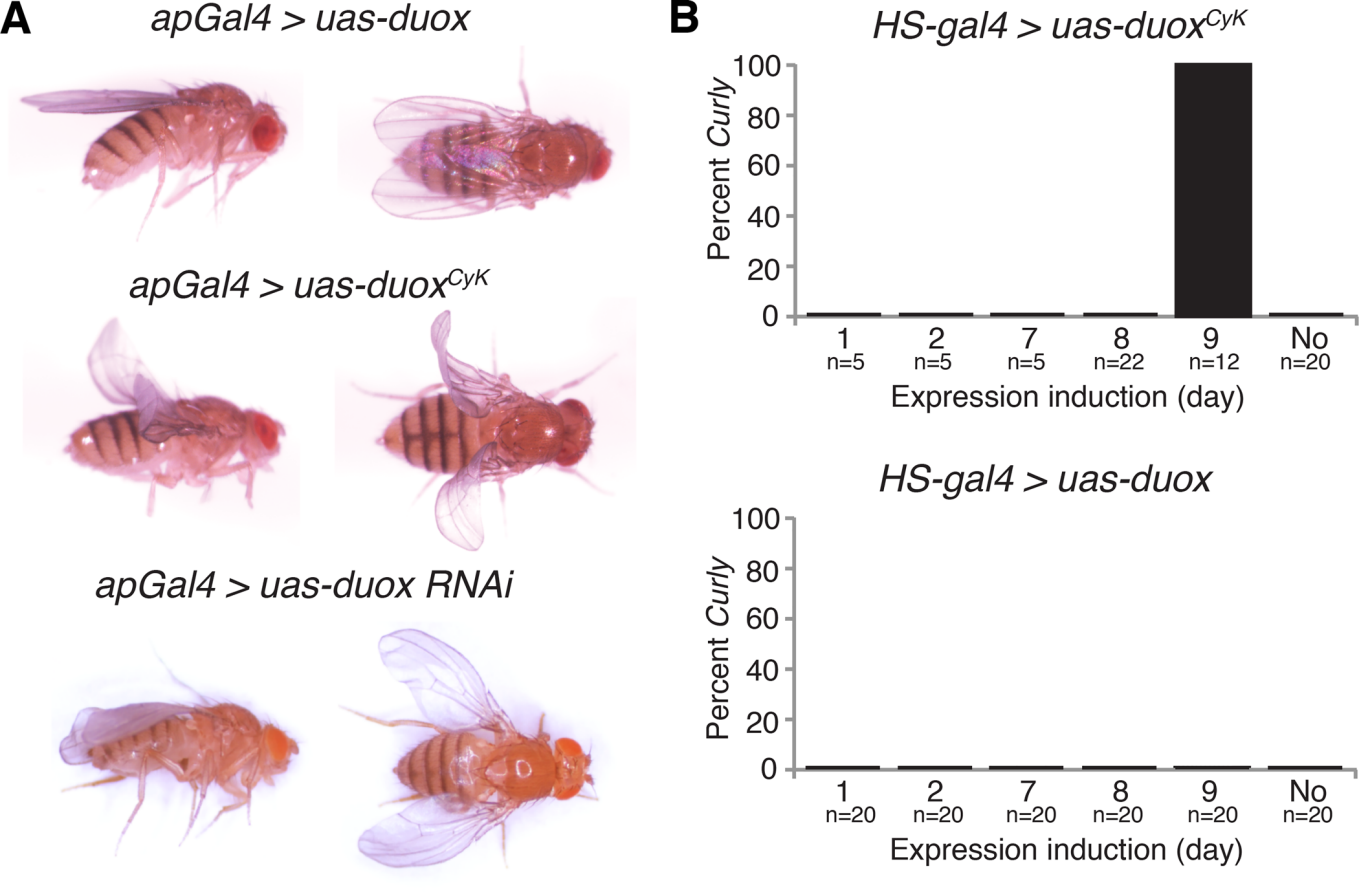
*duox* is required within the wing during the last day of development for wing stabilization. (A) Expression of *duox* ^CyK^, but not *duox*, in the dorsal wing compartment causes a *Curly* phenotype. Knockdown of *duox* in the dorsal wing compartment causes downward cupping and bending of the wing. (B) Expression of *duox* ^CyK^ but not wild-type *duox* on day 9 of development, but not before then, causes a *Curly* phenotype.

Duox could be required autonomously within the wing for its formation, or instead act non-autonomously in other tissues, as is the case for *curled*, a mutation that causes similar changes in wing morphology [2]. To determine where *duox* is required, we first expressed *duox* ^*CyK*^ or *duox* RNAi in the wing using *apGal4*. Expression of *duox* ^*CyK*^, but not wild-type *duox*, caused the upward wing curvature, whereas knockdown of *duox* in the wing caused a slight downward curvature or cupping of the wing (Fig 3A). This demonstrates that *duox* is required autonomously in the wing for its formation.

Changes in wing curvature can be caused by differential growth between the dorsal and ventral wing surfaces, or could be caused by changes in cuticle structure [3, 4]. If *duox* ^*CyK*^ were differentially influencing growth, we would expect it to be required early in pupal development, but not later, when proliferation and growth of the ventral and dorsal wing surfaces is largely complete [18]. To determine when in development *duox* ^*CyK*^ is acting, we conditionally expressed it at various developmental stages using a heat shock-inducible driver. Expression of *duox* ^*CyK*^, but not *duox*, on the last day of pupal development, but not before then, resulted in upturned wings (Fig 3B). This suggests that Duox does not influence wing growth and instead perhaps plays a role in the formation of the wing cuticle. Taken together, our results demonstrate that *duox* acts autonomously in the wing to stabilize the cuticle during the last day of pupal development concurrent with the formation of the wing cuticle.

### Duox works with the heme peroxidase Cysu to stabilize the wing

**Fig 4:**
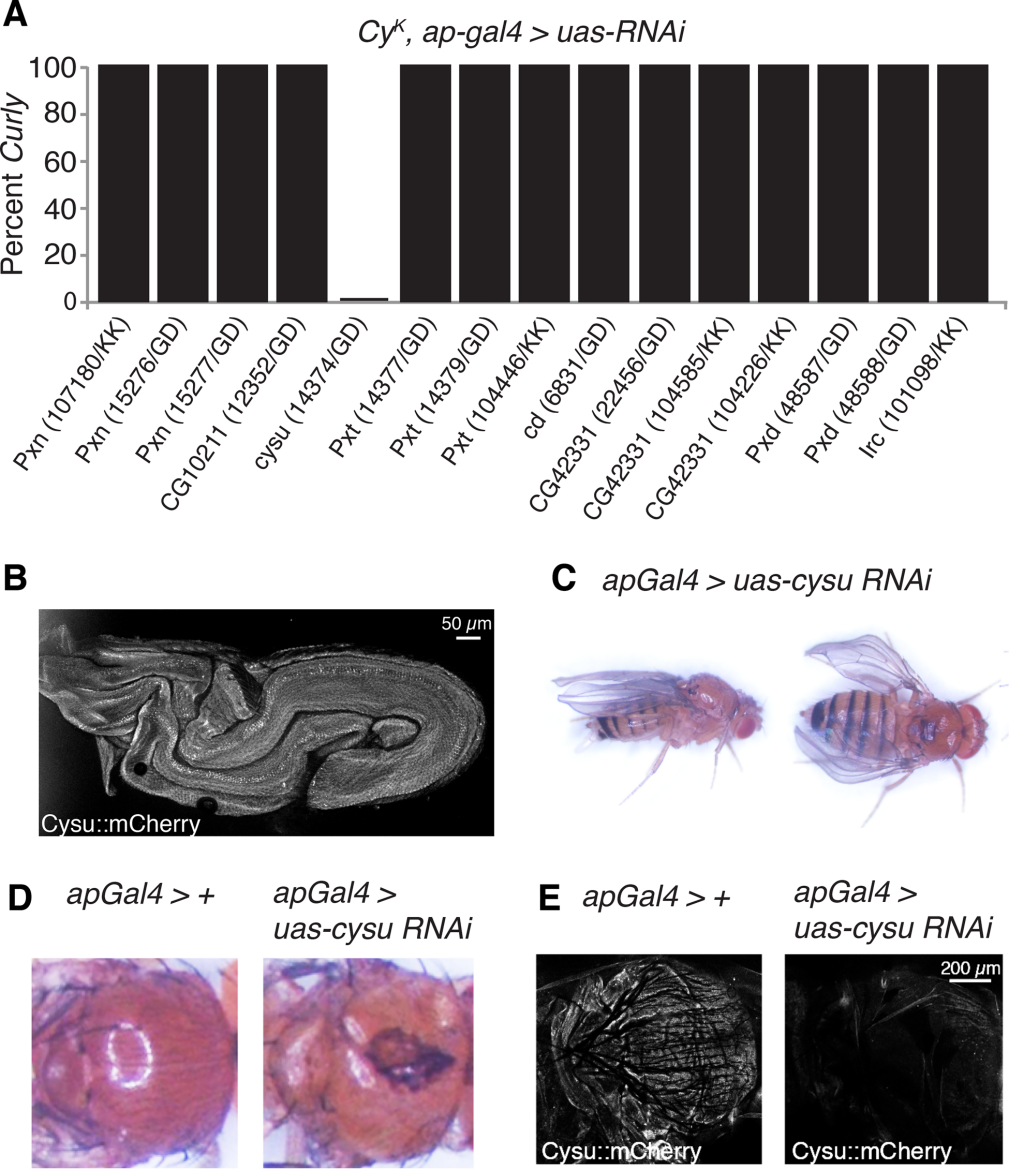
The heme peroxidase Cysu works with Duox to stabilize the wing. (A) Knockdown of *cysu*, but not the other seven *Drosophila* heme peroxidases, suppresses the *Curly* wing phenotype. (B) An endogenously tagged mCherry::Cysu is expressed in the late pupal wing. (C) Knockdown of Cysu in the dorsal wing compartment causes downward cupping and bending of the wing resembling Duox knockdown wings. (D) Knockdown of Cysu with apGal4 in a *Cy* ^*K*^ background causes defects in scutellum and notum formation. (E) Cysu::mCherry is expressed in the thorax of control flies, but not those expressing *cysu* RNAi with apGal4.

Hydrogen peroxide generated by NADPH oxidases is often used by peroxidases, most frequently heme peroxidases, to crosslink proteins and kill pathogens [6]. Heme peroxidases are essential for NADPH oxidase-dependent crosslinking reactions, but largely dispensable to their other functions [6]. To test whether Duox acts with a heme peroxidase in the wing to crosslink proteins and stabilize it, we individually knocked down all known *D*. *melanogaster* heme peroxidases (Fig 4A) [19] using RNAi in the wings of *duox* ^*CyK*^ flies. If *duox* ^*CyK*^ acts alone in the wing and does not participate in crosslinking reactions, then knockdown of heme peroxidases should not affect wing curvature. If, however, *duox* ^*CyK*^ requires a heme peroxidase to function, then knockdown should suppress the *Curly* phenotype. Consistent with this, knockdown of one peroxidase, *CG5873*, fully suppressed the *Curly* phenotype in a manner resembling *duox* knockdowns, suggesting that Duox functions together with CG5873 to crosslink molecules to form the wing (Fig 4A). As CG5873 is a suppressor of *Curly* we have named it *Curly Su*, or *cysu* for short.

If *cysu* acts with *duox* to form the wing then one would expect it to: a) be expressed in the wing on the last day of development–the period in which Duox functions to form the wing; and b) have a similar phenotype to Duox when silenced in the wing. To test this first prediction, we generated a strain expressing endogenously mCherry-tagged Cysu. Consistent with Cysu functioning in the wing, we observed expression of mCherry-tagged Cysu in the wing on the last day of development using confocal microscopy (Fig 4B). To test our second prediction, we knocked down Cysu in the wing using RNAi. Cysu knockdown caused a slight downturning and cupping of the wing resembling Duox knockdown wings, further suggesting that Cysu and Duox function together to shape the wing (Fig 4C). Interestingly, defects in scutellum and notum formation were also observed in Duox and Cysu knockdowns, suggesting that they might play a broader role in cuticle formation (Fig 4D). Consistent with this, Cysu was expressed in the thorax of wild-type flies, but not Cysu knockdowns (Fig 4E). In summary, Duox and the heme peroxidase Cysu act together to stabilize the wing during development.

### Duox is required for adhesion of the dorsal and ventral wing cuticles

**Fig 5:**
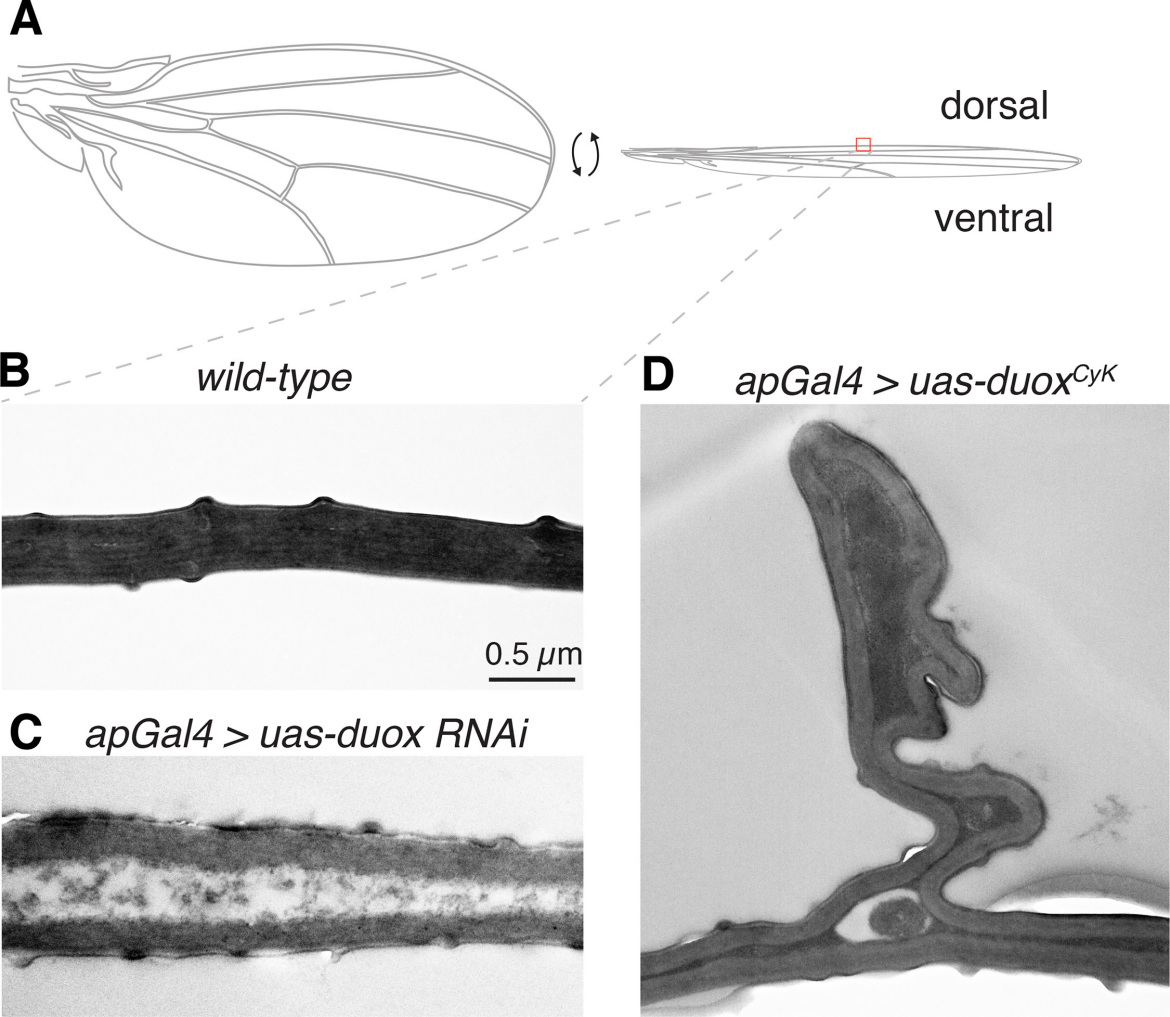
Duox is required for the adhesion and bonding of the dorsal and ventral wing cuticle panel. (A) Cartoon of a wing. (B) In a wild-type wing, the dorsal and ventral cuticles are in close contact. (C) In *duox* knockdown wings, the dorsal and ventral cuticles are infrequently in direct contact. (D) In *Curly* wings, produced by expression of *duox* ^*CyK*^ in the dorsal compartment of the wing with apGAL4, bunching of the dorsal wing surface can be observed.

The *Drosophila* adult wing is made up of two cuticle panels that are synthesized and secreted in a step-wise fashion by the dorsal and ventral ectodermal epithelial cells toward the end of pupal development [20, 21]. Upon eclosion, the dorsal and ventral cuticular surfaces of each wing expand and within an hour or so become bonded and adherent to one another [18]. To determine how altering Duox influences wing cuticle formation, we imaged wild-type and *duox* mutant wings using transmission electron microscopy (Fig 5). In wild-type wings, the ventral and dorsal cuticles are closely apposed and tightly bonded (Fig 5A). By contrast, in Duox knockdown wings the ventral and dorsal cuticles rarely directly contact one another, and instead are separated by a gap filled with disordered, electron poor material (Fig 5B). Interestingly, *Curly* mutant wings on the other hand appeared much more similar to wild-type wings with occasional abnormal bunching of the dorsal cuticle (Fig 5C). Whether this pinching and bunching reduces the surface area of the dorsal wing causing the wing to curve upward remains unknown. However, ultrastructure experiments clearly demonstrate a role for Duox in the adhesion and bonding of the two cuticle wing surfaces during wing formation.

## Discussion

Here we have shown that the *Curly* mutation arises in the NADPH-binding pocket encoding region of *duox*. Using *Curly* mutations and *duox* RNAi, we show that Duox is required within the wing to maintain its shape beginning on the last day of pupal development. Results from our genetic studies suggest Duox does this by supplying hydrogen peroxide to the heme peroxidase Cysu to facilitate the bonding of the two wing cuticle surfaces, likely by physically crosslinking them, during wing formation.

In all *Curly* mutants sequenced, a glycine residue, 1505, in the NADPH-binding pocket of Duox is mutated. This glycine is present in all NADPH oxidases from microbial eukaryotes to humans, and more broadly in oxidoreductase and ferric reductase NAD-binding domains (PFAM PF00175 and PF08030, respectively). Though mutagenesis studies have not been conducted on this residue itself, it sits beside an equally conserved cysteine residue, which has been studied in detail because mutations in it cause chronic granulomatous disease in humans [22]. This cysteine residue does not appear to be important for NADPH oxidase assembly or binding NADPH [22, 23]. Instead it is thought to be required for orienting bound NADPH for efficient electron transfer (via hydride) to FAD, and eventually oxygen [22]. Given glycine 1505’s proximity, it is possible that mutations in it similarly affect the transfer of electrons from NADPH to FAD. Consistent with this is the observation that *Curly* mutants are neither homozygous viable nor viable over a deficiency, suggesting that mutation of glycine 1505 causes a reduction in Duox’s normal function.

Although *Curly* mutations reduce Duox’s normal function, they also endow it with a new function. Precisely what this new function is remains obscure, however it likely requires a source of electrons because altering the NADPH/NADP^+^ by removing niacinamide from the food or knocking down NAD^+^ kinase suppressed the wing phenotype. It is known that the expressivity of the *Curly* wing phenotype can be suppressed by larval crowding and/or starving larvae [24]. Given this, it is possible that reduced uptake of niacinamide is a cause of the decreased expressivity of the *Curly* wing phenotype in starved larvae. Riboflavin shortage during the larval stage has also been suggested to be a cause of this suppression [2, 25]. Since riboflavin is a precursor of FAD, a co-factor also necessary for Duox’s function, it too may suppress the wing phenotype by reducing endogenous FAD and in turn reducing Duox’s activity. Regardless, *Curly* mutations are likely neomorphic and their sensitivity to environmental factors is likely mediated by changes in substrate availability.

Duox is required autonomously for wing stabilization. Results from this study and another strongly support this assertion [10]. Expression of *duox* ^CyK^ or knockdown of *duox* on the last day prior to eclosion, but not earlier, caused defects in wing morphogenesis. This suggests that Duox and *Curly* do not influence growth or proliferation of the wing epithelia because these processes are complete by this time [18]. Instead, ultrastructural analysis suggests that Duox plays an important role in forming the cuticle of the wing. In *duox* knockdowns, frequent gaps between the two wing cuticle surfaces were observed, in contrast to the wild-type wings. Defects in adhesion of the two cuticle surfaces were also apparent in *Curly* mutants. Unlike wings from *duox* knockdowns, however, the cuticle surfaces in *Curly* wings were most often tightly apposed with occasional bunching of the dorsal surface. It is possible that in the *Curly* mutants this aberrant pinching of the dorsal surface decreases its area relative to the ventral surface causing the wing to bend, as first intimated by Waddington 75 years ago [3]. However, we do not know whether this is the cause of the curling or just a consequence of it.

Duox is known to be involved in the formation of extracellular matrices and cuticles [10–12]. Typically, it does this by supplying hydrogen peroxide to heme peroxidases, which use the hydrogen peroxide to perform crosslinking reactions. Consistent with Duox playing a role in crosslinking the cuticle we found that the heme peroxidase Cysu was essential for Duox function in the wing. Duox is unusual among NADPH oxidases in that it contains its own peroxidase homology domain, which in *Caenorhabditis elegans* and *D*. *melanogaster* has been proposed to fulfill the function of heme peroxidases, thereby obviating their need [7, 26, 27]. However, given that the peroxidase homology domain of *Drosophila* Duox lacks many amino acid residues, including the proximal and distal histidines, essential for efficient peroxidase function it is unclear how well it functions in this capacity [27]. Indeed, our results suggest that in *D*. *melanogaster*, Duox requires the heme peroxidase Cysu not only for stabilizing the wing cuticle, but also in the formation of the notum and scutellum. These findings point to a more general role for Duox and Cysu in cuticle formation.

In *Drosophila*, Duox has been intensely studied in the context of host defense and gut immunity. In the gut, Duox is thought to generate ROS to kill pathogens; flies that have reduced Duox activity have increased susceptibility to infection [7, 26]. Upon infection ROS generated by Duox kill pathogens, and possibly signal intestinal epithelial cells to proliferate and renew [7, 28]. Our results, as well as others, demonstrate that Duox is also critical in the formation of cuticle structures and extracellular matrices [10–12]. It is possible that Duox performs a similar function in the *Drosophila* intestine, perhaps by forming extracellular barriers or structures to protect against infection. Indeed, Duox in conjunction with heme peroxidases has been shown to form such barriers in guts of ticks and mosquitos [29, 30]. It would therefore be interesting to explore whether Duox and possibly Cysu are also involved in forming barriers to protect against infection in the *Drosophila* intestine.

Duox is an important protein that has a number of diverse functions, which we are only beginning to understand. *Curly* mutations provide an excellent opportunity to further explore Duox’s functions by identifying unknown interactors and regulators through unbiased genetic suppressor screens. The identification of Cysu through such an approach demonstrates its feasibility and utility. Such approaches will not only tell us about Duox’s function in the wing, but also about its role in immunity and beyond.

## Materials and Methods

### Drosophila husbandry and strains

Flies were propagated in polystyrene vials (28.5 mm diameter) containing cornmeal molasses yeast medium at 25°C. For most crosses 5 virgin females were mated with 3 to 5 males. Fly strains used are shown in Table 1.

**Table 1:** *Drosophila* strains.

Name	Genotype	Reference/source
*duox* ^*KG07745*^	*y[1];P{y[+mDint2]w[BR*.*E*.*BR] = SUPor-P}*	BDSC (16468)
	*Duox[KG07745]/In(2LR)Gla*,*wg[Gla-1]Bc[1]*	
*duox Df*	*Df(2L)C144*,*dpp[d-ho] ed[1]/*	BDSC (90)
	*In(2LR)Gla*,*wg[Gla-1]Bc[1] Egfr[E1]*	
*Cy* ^*1*^	*T(2;3)Scar*, *Scar[1]/Cy[1]*	BDSC (880)
*Cy* ^*K*^	*Cy[K]/In(2LR)Gla*	BDSC (26167)
*act5C-gal4*	y[1]w[*];P{w[+mC] = Act5C-GAL4}17bFO1/TM6B, *Tb[1]*	BDSC (3954)
*apGal4*	*y w; P[w* ^*+*^, *ap-GAL4]/CyO*	J. Treisman lab
*uas-duox*	*y[-]v[-];;attP2-pVALIUM22-duox/TM3 Ser (colony 12042-1-1M)*	This study
*uas-duox* ^*CyK*^	*y[-]v[-];;attP2-pVALIUM22-duox[CyK]/TM3 Ser (colony 12042-4-1M)*	This study
*uas-duox RNAi*	*;UASt-dDuoxIR976-1145/CyO*	Won-Jae Lee
*HS-gal4*	*;hs-gal4/CyO*	R. Lehmann lab
*CG6145 RNAi*	*P{KK106577}VIE-260B*	VDRC (104271/KK)
*Pxn RNAi*	*P{KK108816}VIE-260B*	VDRC (107180/KK)
	*w* ^*1118*^ *; P{GD5987}v15276*	VDRC (15276/GD)
	*w* ^*1118*^ * P{GD5987}v15277*	VDRC (15277/GD)
*CG10211 RNAi*	*w* ^*1118*^ *; P{GD5320}v12352/TM3*	VDRC (12352/GD)
*cysu RNAi*	*w* ^*1118*^ *; P{GD6190}v14374*	VDRC (14374/GD)
*Pxt RNAi*	*w* ^*1118*^ *; P{GD6192}v14377*	VDRC (14377/GD)
	*w* ^*1118*^ *; P{GD6192}v14379*	VDRC (14379/GD)
	*P{KK103684}VIE-260B*	VDRC (104446/KK)
*cd RNAi*	*w* ^*1118*^ *; P{GD3115}v6831/CyO*	VDRC (6831/GD)
*CG42331 RNAi*	*w* ^*1118*^ * P{GD12065}v22456*	VDRC (22456/GD)
	*P{KK110316}VIE-260B*	VDRC (104585/KK)
	*P{KK105949}VIE-260B*	VDRC (104226/KK)
*Pxd RNAi*	*w* ^*1118*^ *; P{GD16228}v48587*	VDRC (48587/GD)
	*w* ^*1118*^ *; P{GD16228}v48588*	VDRC (48588/GD)
*Irc RNAi*	*P{KK106899}VIE-260B*	VDRC (101098/KK)
*cysu*::*mCherry*	*;;Mi{pBS-KS-attB1-PT-SA-SD-2-mCherry}CG5873[MI11428]*	This study
*tubpGal4*	y[1]w[*];P{w[+mC] = tubP-GAL4}LL7/TM3, *Sb[1]*	BDSC (5138)
*uast-duox*	*;uast-duox*	Won-Jae Lee

### Conditional expression of duox and duox^CyK^

*duox* and *duox* ^*CyK*^ were conditionally expressed using a gal4 driver downstream of the heat-shock (HS) protein 70 promoter (HS-gal4). At various times during development *Drosophila* were incubated at 37°C for 2 hours to induce expression.

### duox^KG07745^ (BDSC 16468)

To verify the location of *P{SUPor-P}Duox* ^*KG07745*^ we performed inverse PCR. Consistent with the FlyBase report (FBal0226250) the 3’ flanking sequence was at genomic position 2L:2,826,884. However, unexpectedly, we found the 5’ flanking sequence to be at position 2L:2,755,447. This suggests that *P{SUPor-P}Duox* ^*KG07745*^ contains a deletion that perturbes 17 genes from *Bacc* to *duox*.

### Generation of uas-duox and uas-duox^CyK^ strains

The *duox* open reading frame was amplified from cDNA and cloned into the pVALIUM22 vector [31] between XbaI and EcoRI restriction sites using standard methods. The *Cy* ^*K*^ mutation was generated by site-directed mutagenesis using a QuikChange site-directed mutagenesis kit. Constructs were integrated into the attP2 site on the third chromosome using phiC31 integrase by BestGene.

### Generation of Cysu::mCherry strain

mCherry-tagged Cysu expressing flies were made by BestGene by injecting mimic construct #1315 into Mi{MIC}CG5873^MI11428^ (BDSC 56608) [32].

### DNA sequencing

Genomic DNA was crudely isolated by homogenizing one to two flies in 0.2 mg/ml Proteinase K (Roche MC00079), 10 mM Tris pH 8.0, 1 mM EDTA and 25 mM NaCl and incubating for 25 min at 55°C. Proteinase K was subsequently inactivated by boiling the samples for 5 min. *duox* was then PCR amplified from genomic DNA and sequenced by Genewiz.

### Niacinamide medium

Flies were raised on a defined medium with various concentration of niacinamide from embryo to adult. A solution was prepared as described in Table 2 and the pH was adjusted to 7.0 with NaOH. 20 mg/ml agar yeast culture grade (Sunrise Science Products 1910) was dissolved in the solution by heating before adding 0.4 mg/ml cholesterol (Sigma C3045) and niacinamide (Sigma N0636).

**Table 2:** Niacinamide medium.

Material	Volume	Concentration
H_2_O	to 500 ml	
50x BME amino acid solution (Sigma B6766)	50 ml	5X
L-glutamate potassium salt (Sigma G1149)	500 mg	1.0 mg/ml
Glycine (Sigma G-7126)	200 mg	0.4 mg/ml
Sucrose (Sigma	3.75 g	7.5 mg/ml
Biotin (Sigma B4639)	500 μl 10 mg/ml stock	0.02 μg/ml
B12 (Sigma V6629)	500 μl 14 mg/ml stock	0.028 μg/ml
Choline chloride (Sigma C7527)	37.5 mg	75 μg/ml
Ca-pantothenate (Sigma C8731)	3 mg	6 μg/ml
Pyridoxin-HCl (Sigma P6280)	1.5 mg	3 μg/ml
Riboflavin (Sigma R-0508)	1.2 mg	2.4 μg/ml
Thiamine-HCl (Sigma T1270)	0.75 mg	1.5 μg/ml
Folic acid (Sigma F858)	18 mg	36 μg/ml
MnCl_2_.4 H2O (Sigma M3634)	24 mg	48 μg/ml
MgSO_4_.7 H2O (Sigma M-9397)	123 mg	0.246 mg/ml
K_2_HPO_4_ (P285-500 Fischer)	303 mg	0.606 mg/ml
KH_2_PO_4_ (P288-500 Fischer)	303 mg	0.606 mg/ml
NaCl (Sigma S7653)	6.45 mg	0.0129 mg/ml
CaCl_2_ (Sigma C-5080)	6.45 mg	0.0129 mg/ml
FeSO_4_.6 H2O (Sigma F8633)	11.8 mg	0.0129 mg/ml

### Imaging

Fluorescent images were acquired with a 10X/NA objective on a Zeiss LSM 780 confocal microscope. All other images were obtained with a Zeiss SteREO Discovery.V8 microscope.

### Transmission electron microscopy

For transmission electron microscopy, whole flies were immersed in 95% ethanol briefly to get rid of any air bubbles, decapitated and immersed into fixative containing 4% glutaraldehyde in 0.1M PIPES buffer, pH 7.2 at room temperature for 2 hours, and then overnight at 4°C. Flies were next embedded in 1% agar and post-fixed with 2% osmium tetroxide with 1.5% potassium ferricyanide in 0.1M PIPES buffer for 1 hour then en block stained with 1% uranyl acetate in ddH_2_O at 4°C overnight. Samples were dehydrated with ethanol at room temperature before incubation with propylene oxide and embedment in Spurr resin (Electron Microscopy Sciences, Hatfield, PA). 500nm semi-thin sections were stained with 0.1% toluidine blue to evaluate the area of interest. 60nm ultrathin sections were cut, mounted on formvar coated slotted copper grids and stained with uranyl acetate and lead citrate by standard methods. Stained grids were examined under Philips CM-12 electron microscope (FEI; Eindhoven, The Netherlands) and photographed with a Gatan (4k x2.7k) digital camera (Gatan, Inc., Pleasanton, CA).

## Supporting Information

S1 Fig Expression of *duox* does not rescue the *Curly* wing phenotype. *duox* was expressed ubiquitously in a *Cy* ^*K*^ background using *act5c-gal4*.
(TIF)
