# Correction: *Curly* Encodes Dual Oxidase, Which Acts with Heme Peroxidase Curly Su to Shape the Adult *Drosophila* Wing

**DOI:** 10.1371/journal.pgen.1006285

**Published:** 2016-08-26

**Authors:** Thomas Ryan Hurd, Feng-Xia Liang, Ruth Lehmann

The following information is missing from the Funding section: This work was supported by NIH grant R01/R37HD41900.
