# Supplementary figures and images for: Curly Encodes Dual Oxidase, Which Acts with Heme Peroxidase Curly Su to Shape the Adult Drosophila Wing
Source: PLoS Genet. 2015 Nov 20;11(11):e1005625. doi: 10.1371/journal.pgen.1005625 (PMC4654585; doi:10.1371/journal.pgen.1005625)

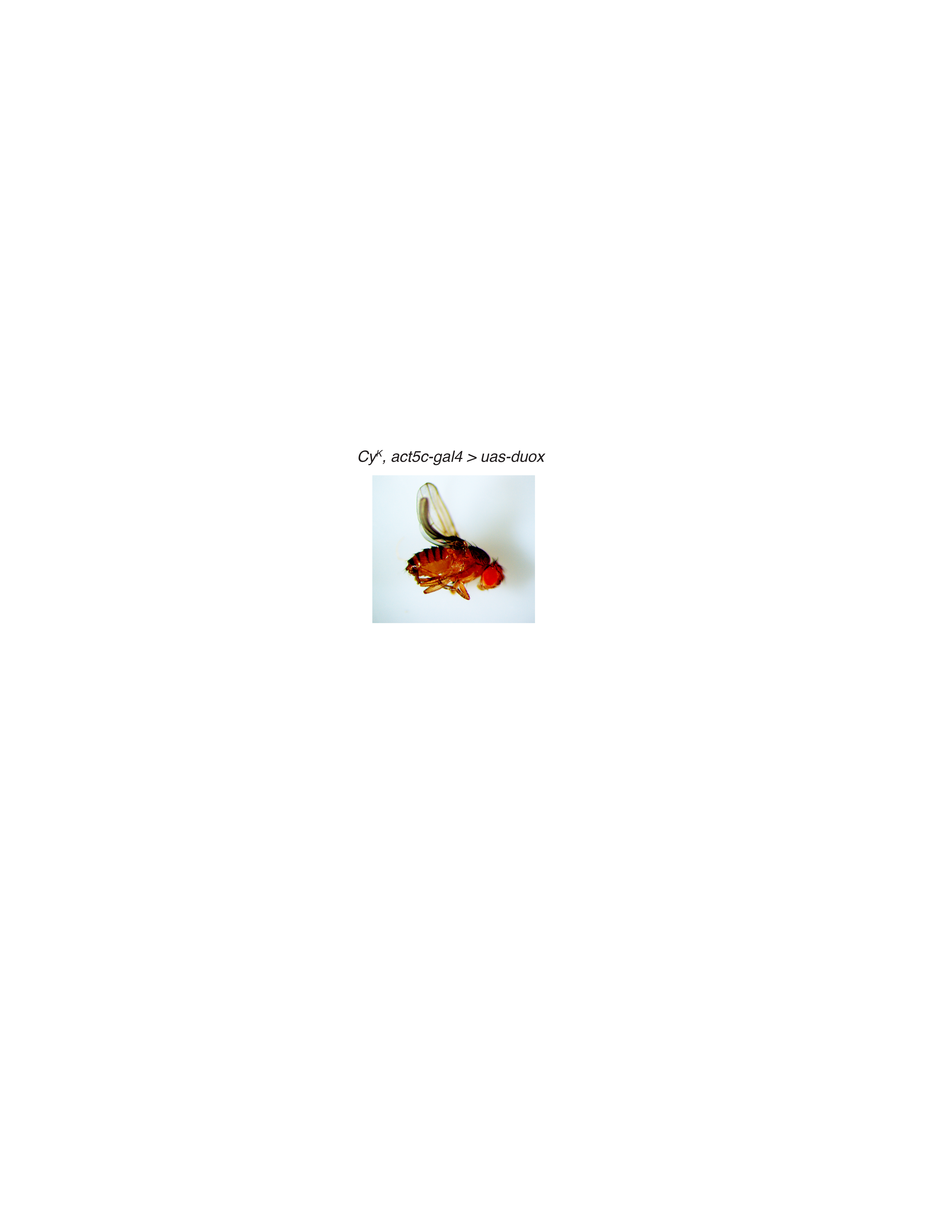

Supplement: S1 Fig — duox was expressed ubiquitously in a Cy K background using act5c-gal4. (TIF) [file pgen.1005625.s001.tif]
